# Subcutaneous Neuromodulation Activates Central Pain‐Suppression Pathways for Analgesia

**DOI:** 10.1002/cns.71004

**Published:** 2026-08-27

**Authors:** Qiuyong Li, Chuan Qin, Can Wang, Jiaqi Lu, Jia Sun, Zhaozheng Li, Zouqin Huang, Sheng Liu

**Affiliations:** ^1^ Shanghai University of Traditional Chinese Medicine Shanghai China; ^2^ Shanghai Pudong New Area Hospital of Traditional Chinese Medicine Shanghai China

**Keywords:** analgesia, neural pathway, nucleus accumbens, pain, subcutaneous mechanical stimulation

## Abstract

**Aims:**

Peripheral neuromodulation, which can be considered a flow of signals from the body to the brain, induces pain relief. However, whether subcutaneous neuromodulation, which regulates specific neural circuits for analgesia, remains largely elusive. This study aimed to evaluate the analgesic effects of subcutaneous mechanical stimulation (SMS) on neuropathic pain.

**Methods:**

A stainless‐steel needle (0.5 cm long) was inserted into the subcutaneous tissue. Mechanical allodynia, thermal hyperalgesia, and conditioned place preference (CPP) were assessed in rodent models of localized inflammation of the dorsal root ganglia (LID) and spared nerve injury (SNI). Optogenetic and chemogenetic tools were employed in conjunction with electrophysiological recordings, molecular analyses, and pharmacological interventions to elucidate the underlying mechanisms of SMS‐induced analgesia.

**Results:**

Subcutaneous needle insertion generated mechanical force, activating Piezo channels. SMS attenuated mechanical allodynia and thermal hyperalgesia and induced CPP in the LID and SNI models. Naloxone pretreatment did not significantly affect SMS‐induced analgesia. SMS activated GABAergic neurons in the NAc shell, and their inhibition reversed the SMS effects in LID rats. Furthermore, activation of the IL‐to‐nucleus accumbens (NAc) shell projections was essential for SMS‐induced analgesia and CPP.

**Conclusion:**

These findings indicate that SMS activates central pain‐suppression pathways, providing a potential framework for clinical pain management.

## Graphical abstract

Subcutaneous neuromodulation (SMS), as a novel neuromodulatory approach different from traditional thermal or electrical methods, modulates peripheral‐to‐central neural signaling and induces rapid, opioid‐independent analgesia. SMS exerts strong analgesic effects by activating the IL to NAc pathway, a key neural circuit involved in top‐down pain modulation.

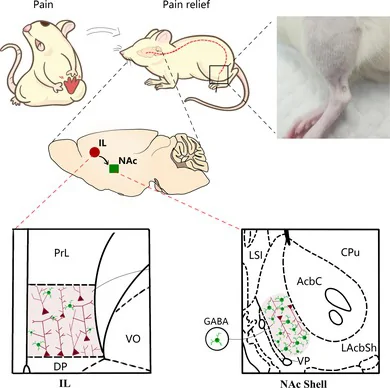

## 1 Introduction

Peripheral nerve stimulation (PNS) has become an innovative approach in modern medicine, significantly advancing the diagnosis and management of complex disorders in recent decades [1, 2]. It involves the application of electrical impulses to modulate organ activity or alleviate pain, typically requiring surgical implantation of electrodes in peripheral regions, such as the limbs or spinal cord. However, the invasive nature of this procedure carries significant risks, including surgical complications, local discomfort, tissue injury, and inflammation [3, 4]. Noninvasive alternatives, such as transcutaneous electrical nerve stimulation (TENS) and electroacupuncture (EA), have been developed to overcome the limitations of invasive procedures. TENS delivers low voltage electrical currents through the skin, whereas EA involves inserting and manipulating needles at acupuncture points to activate peripheral nerve endings [5]. However, these approaches also have inherent limitations. TENS devices are often nonportable and constrain therapy to brief sessions [6], whereas EA can elicit discomfort, including soreness, numbness, heaviness, or distension, potentially reducing patient compliance.

The therapeutic efficacy of the PNS critically depends on the anatomical accuracy of the stimulation site [7]. The subcutaneous tissue, the deepest layer of the skin, represents a key anatomical target. It comprises adipocytes and connective tissue matrices that structurally support vascular networks, neural pathways, and glandular components while providing biomechanical linkages between the dermis and underlying musculoskeletal structures [8]. This histologically complex compartment contains specialized neural structures, including mechanosensory receptors, signal‐amplifying nerve terminals with axonal specializations, and transduction cells that interface with diverse subcutaneous neural networks. Peripheral nerve fibers traverse this connective tissue matrix, branching horizontally to form complex subcutaneous plexuses [9]. Despite its elaborate neuroanatomical organization, the subcutaneous layer remains largely underused as a target for neuromodulatory interventions to maintain homeostasis or modulate pathophysiological states [10].

This study aimed to establish subcutaneous mechanical stimulation (SMS) as a novel neuromodulatory strategy different from thermal or electrical modalities. SMS is capable of eliciting rapid, opioid‐independent analgesia by modulating peripheral‐to‐central neural signaling. This investigation further sought to identify the involvement of the infralimbic cortex (IL) in the nucleus accumbens (NAc) pathway in SMS‐induced pain suppression, with the aim of evaluating the subcutaneous layer as a potential therapeutic target for clinical neuromodulatory applications.

## 2 Materials and Methods

### 2.1 Animals

Adult Sprague–Dawley rats (8–10 weeks old, 250–300 g; SLAC) and male C57BL/6J mice (6–8 weeks old; SLAC) were used in this study. The rationale for using male animals in this initial mechanistic study is as follows: (1) The estrous cycle in females has a well‐documented influence on pain sensitivity and neuronal excitability, which introduces considerable variability that could confound the interpretation of the SMS mechanism under investigation [11]. (2) The use of a single sex helps minimize variability and enhances internal consistency in this preliminary study—an approach supported by the literature on establishing novel pain models [12].

All animals were obtained from the Laboratory Animal Center of Shanghai University of Traditional Chinese Medicine.

### 2.2 Spared Nerve Injury (SNI)

As previously described, surgical procedures were performed under 5% isoflurane anesthesia [13]. A longitudinal incision was made along the lateral aspect of the right femur, followed by blunt dissection of the biceps femoris muscle to expose the sciatic nerve trifurcation. Distal to this point, the common peroneal and tibial nerve branches were isolated, double‐ligated via 6–0 surgical silk sutures, and excised to remove 2 mm nerve segments while preserving the integrity of the sural nerve.

### 2.3 Localized DRG Inflammation (LID)

As previously described [14], inflammatory radiculopathy was induced by administering zymosan A to the dorsal root ganglia (DRG). Under isoflurane anesthesia, a 0.5‐in. 30‐gauge needle was inserted perpendicularly into the L4 intervertebral foramen in the mice or L5 in the rats. A sterile solution of zymosan A (2 mg/mL in saline, 10 μL) was injected to induce localized DRG inflammation. Sham‐operated controls underwent identical surgical procedures, including DRG exposure, but without injection.

### 2.4 SMS

To accommodate the anatomical scale of the animals, a stainless‐steel needle (0.08 mm diameter × 5 mm length) was fabricated by modifying a standard dispensing needle (0.08 mm diameter × 15 mm length). The needle was inserted 5 mm lateral to the achilles tendon and 7 mm above the ankle joint in rats (3 mm for mice), and needle‐holding forceps were used for precise placement. Initial insertion was performed at a 30° angle to the skin surface, followed by advancement parallel to the dermal plane. The needle was secured in place via medical tape. In the sham control groups, needles were positioned adjacent to the skin without penetration and were similarly fixed with tape to ensure procedural consistency [15]. The needle was left in place for 20 min. The SMS stimulation site is placed within the sural nerve's cutaneous distribution territory, which remains fully innervated. Therefore, SMS is applied to a region that retains functional sensory input, allowing for mechanotransduction via intact sural nerve afferents.

### 2.5 Conditioned Place Preference (CPP)

CPP was performed as described previously [16]. A standard two‐chamber balanced design was used. On the preconditioning day, animals were allowed to travel the chambers freely for 15 min, and the amount of time spent in each chamber was recorded. Rats that spent > 720 s of the total time (900 s) on one side were eliminated from subsequent procedures. In fact, none of the animals had a substantial initial bias for either compartment in our experiment. Next, two conditioning sessions (for SMS and gentle handling) were performed for three consecutive days. Animals were treated with either SMS or gentle handling for 30 min each in the morning and afternoon. We immediately placed the animals into the pairing chamber and confined them to the chamber for 30 min after SMS administration or gentle handling. For every conditioned animal, SMS and gentle handling were carried out alternatively in the morning and afternoon sessions. The morning and afternoon SMS stimulation and gentle handling were conducted at least 4 h apart. A preference test was conducted 3 days after conditioning. Animals were placed in the CPP apparatus with a door opened in the middle for 15 min to allow access to the apparatus's whole parts in this phase. The preference scores were analyzed by calculating the amount of time the animals spent in the SMS paired chamber minus the time they spent in the gentle handling chamber.

### 2.6 Tissue Preparation and Scanning Electron Microscopy (SEM)

Details of the SEM method are provided in the appendix materials. Briefly, the fixed hindlimb tissue was excised and placed under a dissecting microscope (Leica M165FC). A sterile disposable blade was used to make longitudinal incisions parallel to the needle shaft and perpendicular to the skin surface, starting 5 mm from the needle insertion site [17].

### 2.7 Immunofluorescence Staining

Tissue section staining was performed according to previously established protocols [18]. Briefly, the sections were washed three times with PBS, blocked with 1% BSA for 2 h at 4°C, and incubated with primary antibodies for 48 h at 4°C. After three additional PBS washes, the sections were incubated with the appropriate fluorescent secondary antibody (1:500) for 2 h. The sections were then mounted on adhesive slides with coverslips via fluorescence decay‐resistant mounting medium. Imaging was performed via a Leica laser scanning confocal microscope.

### 2.8 Cannula Surgeries and Microinjection

Thirty minutes before SMS, the rats received bilateral microinjections into the NAc shell [19]. The obturators were removed from the guide cannulas, and a 33‐gauge injector needle (42‐0210; Hamilton, Bonaduz, Switzerland) was inserted such that the tip extended 2 mm beyond the end of the cannula. The animals were administered either bicuculline (Bic; 3.0 nmol/0.3 μL, 10 mM in DMSO; MCE, HY‐N0219, Monmouth Junction, NJ, USA) or vehicle control (0.3 μL of DMSO; Sigma–Aldrich, D2650). The infusions were delivered at a rate of 0.3 μL/min via a microinfusion pump (CMA 100; Carnegie Medicine), and the injector was left in place for an additional 3 min post infusion to facilitate diffusion.

### 2.9 Optogenetic Manipulation

Implantable optic fibers (200 μm diameter, 0.37 NA; Doric Lenses) were connected to a 473 nm blue laser system (Cube 473‐100; Cobolt) through optical fiber couplers (Doric Lenses). The stimulation parameters were controlled via a Doric NeuroStar interface, which delivers light at 6 mW power, 10 ms pulse width, and 10 Hz frequency continuously for 30 min. The same fiber‐coupled system was used without light emission in the control groups to ensure procedural consistency. Optogenetic manipulations were conducted during the conditioning phase of the CPP paradigm. Mice with off‐target viral expression or incorrect optic fiber placement, as confirmed by post hoc cresyl violet staining, were excluded from the analysis [20].

### 2.10 Statistical Analysis

Statistical analyses were performed via GraphPad Prism 8.0 (GraphPad Software) and TBtools [21]. The data are presented as the mean ± SEM. All continuous data were first assessed for normality using the Shapiro–Wilk test. Data conforming to a normal distribution were analyzed using parametric tests. Paired or unpaired Student's *t*‐tests were used for comparisons between two groups, depending on the experimental design. For normally distributed data, one‐way ANOVA or two‐way repeated‐measures ANOVA was conducted, followed by post hoc comparisons using Tukey's test (for one‐way ANOVA) or Bonferroni (for two‐way repeated‐measures ANOVA). Data that were not normally distributed were analyzed using the Mann–Whitney *U* test. The level of statistical significance was *p* < 0.05, with exact *p* values reported for all comparisons (shown in Figure legends).

## 3 Results

### 3.1 Stimulation Site, Needle Insertion Parameters, and Sensory Characteristics of SMS

**FIGURE 1:**
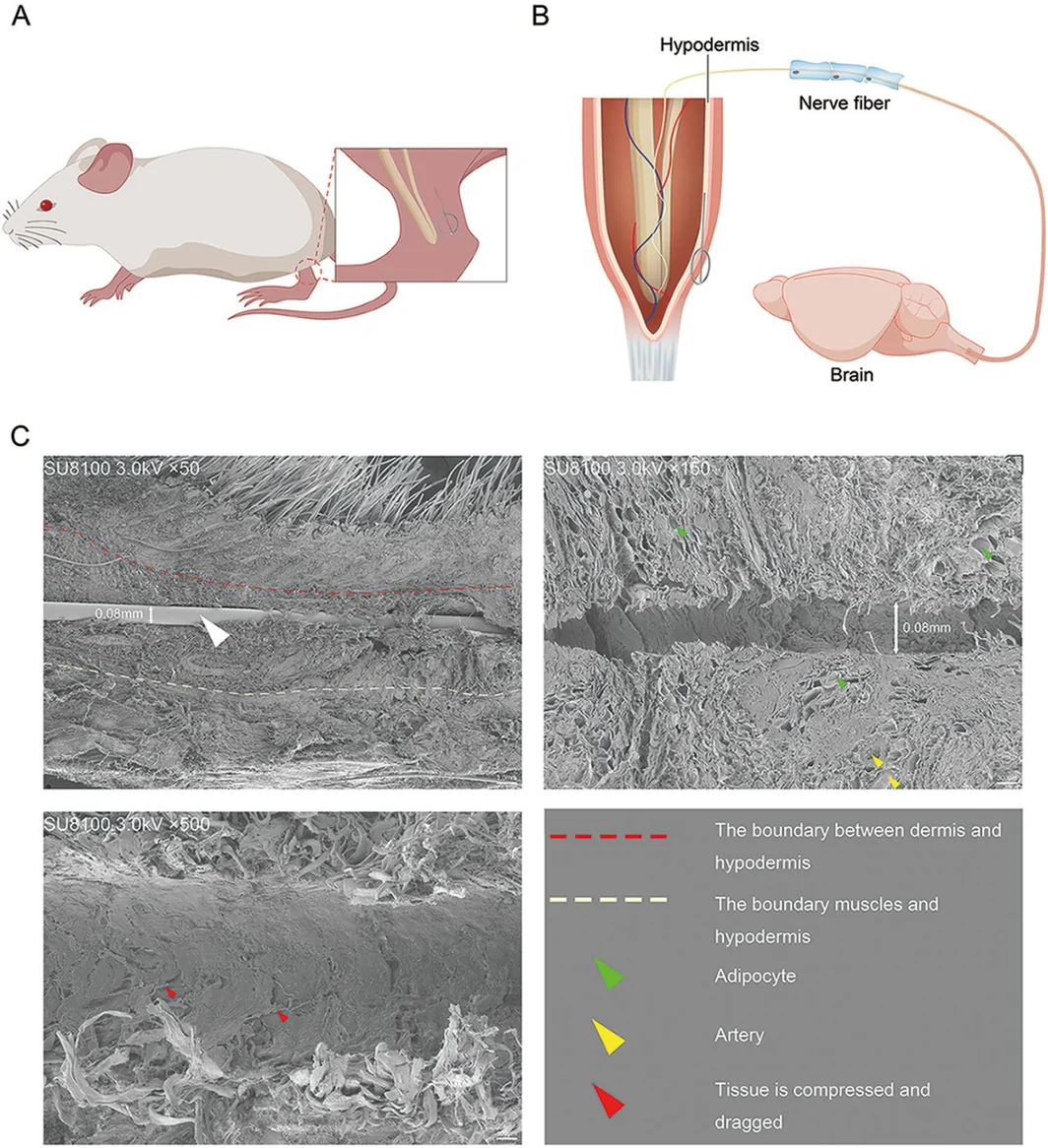
Subcutaneous mechanical stimulation mode (A) Schematic of subcutaneous mechanical stimulation in rats. (B) Schematic of the depth of subcutaneous mechanical stimulation at the skin level. (C) Scanning electron micrograph of subcutaneous mechanical stimulation (the red dotted line separates the muscle from the subcutaneous connective tissue; the white dotted line marks the boundary between the dermis and the subcutaneous connective tissue.).

As illustrated in Figure 1, the anatomical layers beneath the stimulation site include the skin, subcutaneous tissue, peroneus brevis muscle, and flexor hallucis longus muscle. This region lies within the innervation territory of the sural nerve. The skin in this area is supplied by the lateral sural cutaneous nerve, a branch of the common peroneal nerve originating in the popliteal fossa. The stimulation site was sterilized before the procedure to prevent contamination during the procedure. Experimental observations indicated that the tactile stimulation produced as the needle penetrates the skin elicited the transient hindlimb withdrawal response (Video S1). Rats in the sham SMS group (in which the needle touches but does not penetrate the skin) also displayed withdrawal responses (Video S2). In addition, SMS‐treated rats exhibited increased general activity along with fewer withdrawal responses and less paw‐licking behavior (Video S3). Given the short needle length (0.5 cm), the penetration was presumed to be limited to subcutaneous tissue and did not extend into the muscularis propria. This stimulation modality involves a single needle insertion without further manipulation. Clinical observations have indicated that it evokes only a mild sensation comparable to tactile skin stimulation without producing the characteristic pinprick sensation typically associated with invasive procedures [22, 23, 24].

### 3.2 SMS Produces Opioid‐Independent Analgesia and CPP in the LID and SNI Models

**FIGURE 2:**
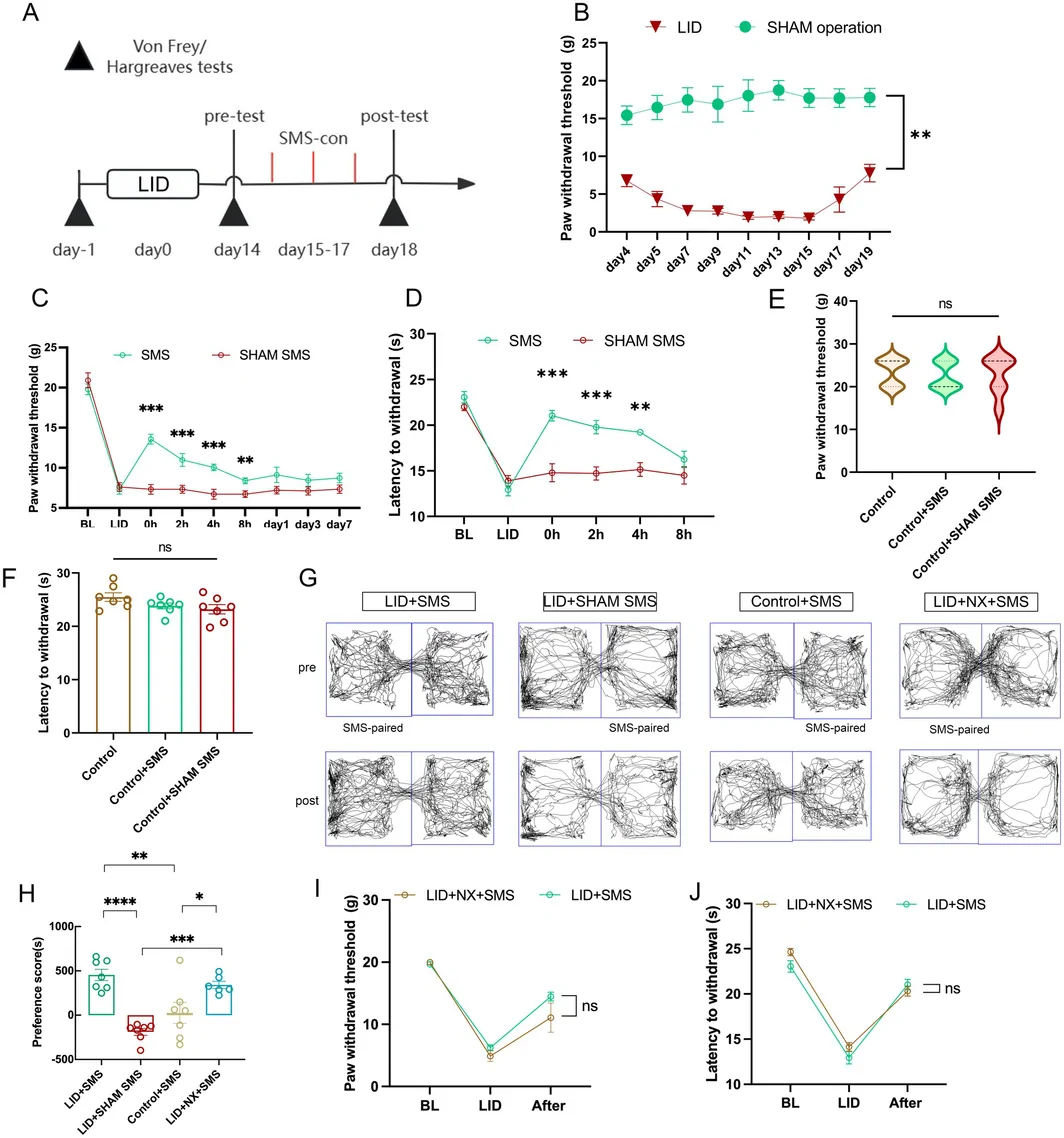
SMS induced analgesia and CPP in rats with LID (A) Schematic showing the experimental protocol. (B) Paw withdrawal threshold curve of rats after the LID model (*n* = 6 in each group; two‐way RM ANOVA was used for data analysis; *F* (8, 80) = 2.585; Day 13, LID vs. Sham operation ****p < 0.0001, **p < 0.01). (C) Paw withdrawal threshold of the rats at baseline, in the LID model, and at different times after SMS (*n* = 16 in the SMS group, *n* = 13 in the SHAM SMS group; a mixed‐effects model (REML) was used for data analysis; *F* (8, 179) = 8.0, 0 h, LID+SMS versus LID+SHAM SMS ***p < 0.001; 2 h, LID+SMS vs. LID+SHAM SMS ***p < 0.001; 4 h, LID+SMS versus LID+SHAM SMS ***p < 0.001; 8 h, LID+SMS versus LID+SHAM SMS **p < 0.01). (D) Rats received SMS on day 14 after LID. SMS attenuated immediate pain hypersensitivity, as indicated by latency to withdrawal (*n* = 7 in each group; two‐way RM ANOVA was used for data analysis; *F* (5, 60) = 9.665, 0 h, LID+SMS versus LID+SHAM SMS ***p < 0.001; 2 h, LID+SMS vs. LID+SHAM SMS ***p < 0.001; 4 h, LID+SMS vs. LID+SHAM SMS **p < 0.01). (E) Effect of SMS on the paw withdrawal threshold in control rats (*n* = 4 in each group). (F) Effect of SMS on the latency to withdrawal in control rats (*n* = 4 in each group). (G) Representative activity trajectories of real‐time movement traces among the groups during the CPP test. (H) CPP scores of the animals in each group (*n* = 7 in the LID+SMS, LID+SHAM SMS, and Control+SMS groups; *n* = 9 in the LID+NX + SMS group; one‐way ANOVA was used for data analysis; *F* = 8.604; LID+SMS versus LID+SHAM SMS ****p < 0.0001; LID+SMS versus Control+SMS **p < 0.01; LID+SHAM SMS versus LID+NX + SMS ***p < 0.001; Control+SMS versus LID+NX + SMS *p < 0.05). (I) Effect of SMS on the paw withdrawal threshold in LID rats after naloxone intraperitoneal injection (*n* = 9 in the LID+SMS group, *n* = 7 in the LID+NX + SMS group; two‐way RM ANOVA was used for data analysis; *F* (4, 42) = 11.24, LID+SMS versus LID+NX + SMS *p* > 0.05). (J) Latency to withdrawal of LID rats after naloxone intraperitoneal injection (*n* = 9 in the LID+SMS group, *n* = 7 in the LID+NX + SMS group; two‐way RM ANOVA was used for data analysis; *F* (4, 42) = 10.77, LID+SMS versus LID+NX + SMS *p* > 0.05).

Rat models of local ischemic injury (LID) and sciatic nerve injury (SNI) were established and subjected to SMS treatment at designated time points (Figure 2). Nociceptive thresholds were evaluated via the Von Frey and Hargreaves tests, whereas emotionally motivated behaviors were assessed via CPP testing (Figure 2A). On postoperative day 14, the paw withdrawal threshold in the LID group was significantly lower than that in the sham‐operated group (Figure 2B). Compared with sham‐operated control rats, LID rats receiving ipsilateral SMS presented significantly elevated paw withdrawal thresholds at 0, 2, 4, and 8 h posttreatment, with the analgesic effect progressively declining over time (Figure 2C). Consistently, thermal stimulation assays demonstrated a significant increase in paw withdrawal latency at 0, 2, and 4 h following SMS, supporting the presence of a rapid antinociceptive effect in LID rats (Figure 2D). SMS did not alter nociceptive thresholds in normal rats (Figure 2E,F) but produced analgesic effects exclusively in nerve injury models (LID/SNI), indicating that its therapeutic efficacy is dependent on the presence of pain. In CPP testing, SMS elicited a significant preference for the SMS‐paired chamber, an effect that was absent in animals subjected to sham stimulation (Figure 2G). Similarly, in the SNI model, SMS alleviated paw withdrawal threshold and latency to withdrawal and induced CPP (Appendix S1A–H). In female rats with SNI, SMS also relieved mechanical allodynia and thermal hyperalgesia. The effects of SMS‐induced analgesia were maintained for at least 90 min (Appendix S2). Compared with the sham SMS group, SMS‐treated rats exhibited increased general activity, as well as fewer withdrawal responses, less paw‐licking and grooming (Appendix S3A–F).

The potential role of the opioid system in SMS‐induced analgesia was also assessed. Previous literature indicates that 4.0 mg/kg naloxone hydrochloride can effectively antagonize morphine‐induced antinociception [25]. Notably, naloxone 15 min before receiving SMS failed to block SMS‐induced CPP (Figure 2G–J), suggesting that SMS may exert its analgesic effects through an opioid‐independent mechanism.

### 3.3 SMS Analgesia Involves Piezo Activation and Supraspinal Pathways

**FIGURE 3:**
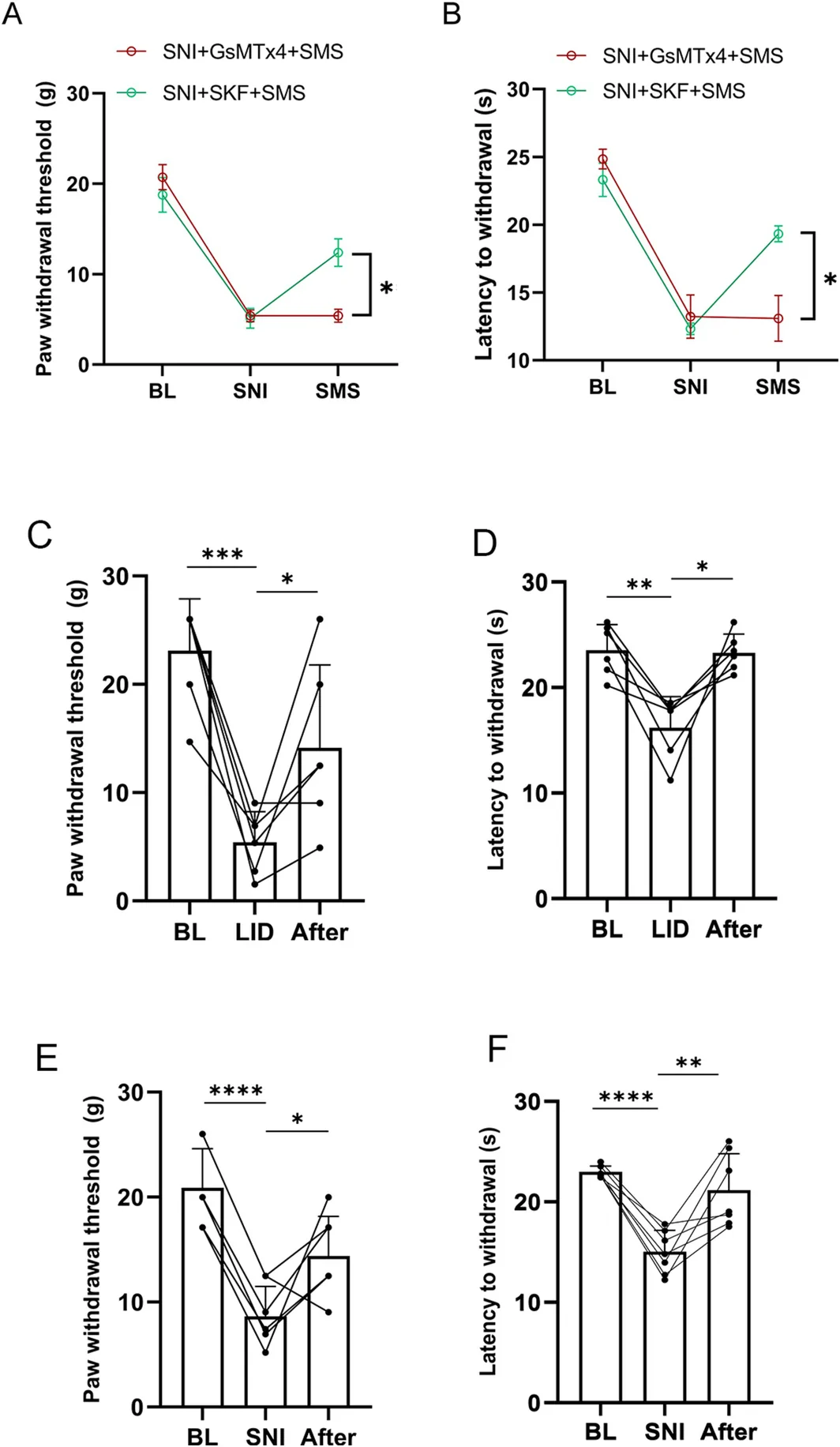
SMS analgesia involves piezo activation and supraspinal pathways (A) Paw withdrawal threshold of SMS stimulation after local injection of GsMTx4 or SKF into SNI‐injured nerves (*n* = 5 in each group; two‐way RM ANOVA was used for data analysis; *F* (2, 16) = 17.57, *p < 0.05). (B) Latency to withdrawal of SMS stimulation after local injection of GsMTx4 or SKF into SNI‐injured nerves (*n* = 5 in each group; two‐way RM ANOVA was used for data analysis; *F* (2, 16) = 17.68, *p < 0.05). (C) Effect of contralateral SMS on the paw withdrawal threshold of LID rats (*n* = 9 in this group, repeated measures ANOVA was used for data analysis, *F* = 15.8, LID versus BL ***p < 0.001, LID versus After *p < 0.05). (D) Effect of contralateral SMS on the latency to withdrawal in LID rats (*n* = 9 in this group, repeated measures ANOVA was used for data analysis, *F* = 13.6, LID versus BL **p < 0.01, LID versus After *p < 0.05). (E) Effect of contralateral SMS on the paw withdrawal threshold in SNI rats (*n* = 9 in this group, repeated measures ANOVA was used for data analysis, *F* = 23.17, SNI versus BL ****p < 0.0001, SNI versus After *p < 0.05). (F) Effect of contralateral SMS on the latency to withdrawal in SNI rats (*n* = 9 in this group, repeated measures ANOVA was used for data analysis, *F* = 22.07; SNI versus BL ****p < 0.0001; SNI versus After **p < 0.001).

Needle insertion into the subcutaneous tissue initiates a mechanical force response. Piezo channels and transient receptor potential (TRP) channels serve as key mechanotransporters across various cell types, facilitating the conversion of mechanical stimuli into electrochemical signals [26, 27, 28]. To assess their involvement in SMS‐induced analgesia, local pharmacological inhibition was performed via the use of GsMTx4 to block Piezo channels and SKF to inhibit TRP channels. Local administration of GsMTx4 significantly reduced the SMS‐induced increase in paw withdrawal thresholds and latency to withdrawal in SNI rats (Figure 3A,B), whereas SKF had no significant effect on SMS‐mediated paw withdrawal thresholds and latency to withdrawal relief (Figure 3A,B). These results suggest that Piezo channels, but not TRP channels, are essential mediators of SMS‐induced mechanical analgesia, supporting the classification of SMS as a form of mechanical stimulation. However, it should be noted that SKF 96365 is a nonselective TRP channel antagonist with known off‐target effects at concentrations used to block TRP channels. Therefore, while our data do not support a primary role for TRP channels in SMS‐induced analgesia, further studies using more selective inhibitors or genetic approaches are warranted to confirm this conclusion.

SMS was delivered to the side opposite the injury to further evaluate whether SMS applied to the contralateral limb modulates neuropathic pain. This contralateral stimulation significantly alleviated nociceptive hypersensitivity in both the LID and SNI models (Figure 3C–F), indicating the involvement of supraspinal structures in mediating the therapeutic effects of SMS.

### 3.4 SMS Activates GABAergic Neurons in the NAc Shell in LID Rats

**FIGURE 4:**
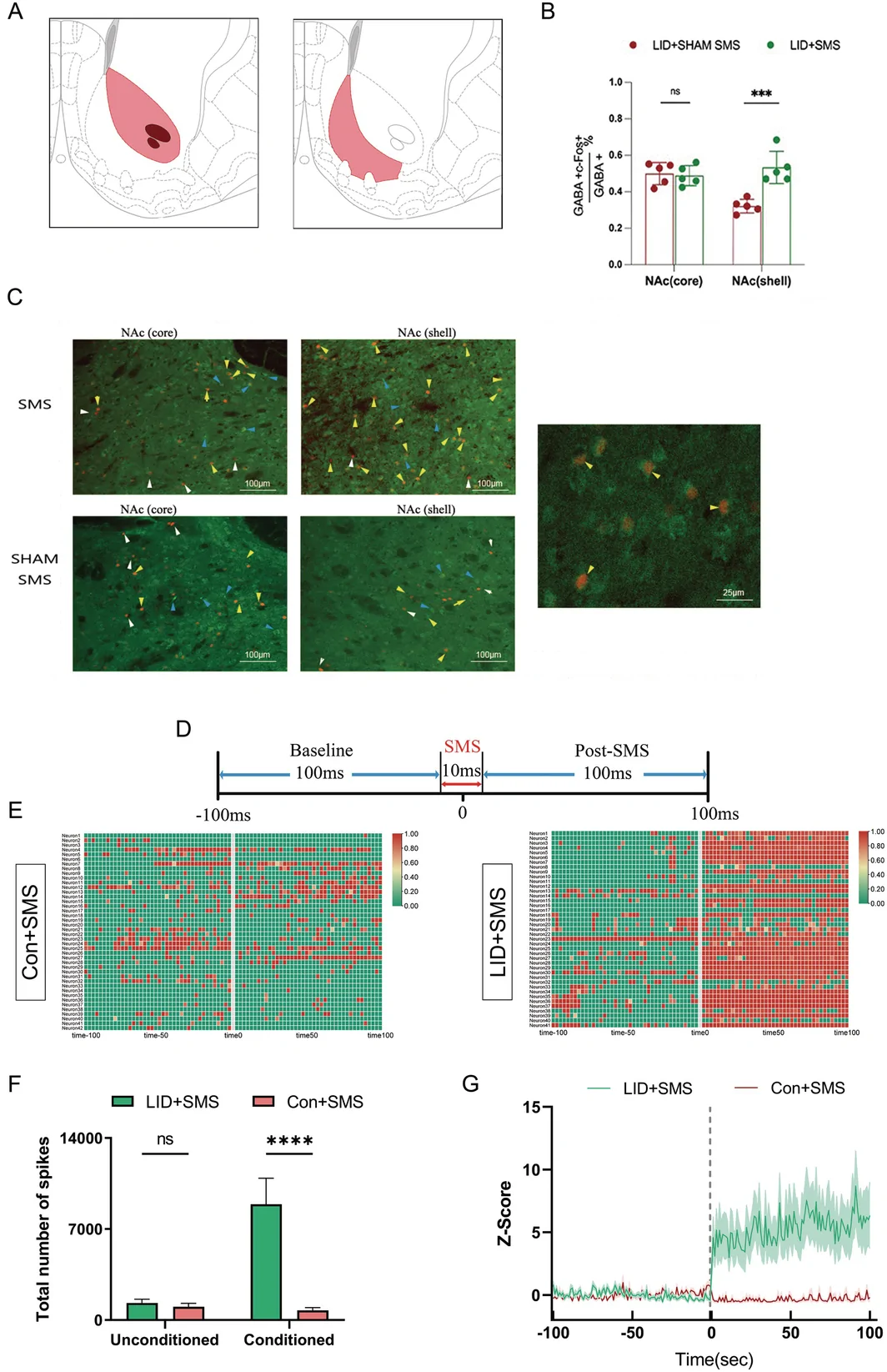
SMS activates GABAergic neurons in the NAc shell in rats with pain. (A) Schematic diagram of the core and shell regions of the nucleus accumbens. (B) The percentages of GABA neurons that were observed to be c‐Fos positive in the core of the nucleus accumbens and shell (*n* = 5 rats in each group; paired *t*‐tests were used for data analysis; ***p < 0.001). (C) Representative photomicrograph showing GABA/c‐Fos immunoreactivity in the NAc core and shell. Representative c‐Fos^+^/GABA^+^ (yellow arrows), c‐Fos^+^/GABA^−^ (white arrows), and c‐Fos^−^/GABA^+^ (blue arrows) cells. Representative double‐labeled GABA^+^/c‐Fos^+^ neurons in the NAc Shell (Right), ×40 magnification. Red: C‐Fos; Green: GABA; Merge (yellow): Colocalization of c‐Fos^+^ nuclei within GABA^+^ neurons. (D) Timeline of recording. SMS was applied over a 10‐s window at time 0, with electrophysiological data aligned to this event. Baseline activity was recorded for 100 s prior to SMS, and post‐SMS activity was analyzed in 100‐s bins thereafter. (E) All recorded energy spectra of GABAergic neurons in the NAc (LID+SMS, neurons *n* = 42 from 5 rats; Con+SMS, neurons *n* = 42 from 5 rats). (F) Total number of spikes distributed per group of animals (LID+SMS, spike *n* = 22 from 5 rats; Con+SMS, spike *n* = 20 from 5 rats; data were compared via two‐way ANOVA. *F* (1, 40) = 12.79, ****p < 0.0001). (G) Mean *z* score activity for NAc putative GABA neurons before (−100–0 s) and after SMS stimulation (0–100 s) in LID rats or control rats, displayed as the mean ± SEM; −100–0 is the unconditioned side, and 0–100 is the conditioned side. The data were compared via two‐way ANOVA, *F* (1, 40) = 9.668.

Previous studies have emphasized the role of the NAc shell in mediating pain, affective–motivational processing, and the salience of external stimuli [29, 30, 31]. Moreover, GABA has been shown to inhibit pain transmission along specific neural pathways [32, 33], suggesting its involvement in descending pain modulation. To assess whether GABA signaling in the NAc shell contributes to the effects of SMS, the protein expression of GABA was examined in SNI rats via c‐Fos/GABA double labeling (Figure 4A). SMS significantly increased c‐Fos expression in GABAergic neurons within the NAc shell (Fos^+^/GABA^+^ neurons: 53% ± 9% in the LID + SMS group versus 32% ± 7% in the LID + Sham group) on Day 14 post‐SNI (Figure 4B,C). No significant changes were observed in the percentage of c‐Fos^+^/GABA^+^ neurons in the NAc core (Figure 4C). These findings indicate that SMS selectively activates GABAergic neurons in the NAc shell but not in the NAc core.

Next, the neuronal activity of NAc shell GABAergic neurons was assessed via multichannel unit recordings before and after SMS in LID rats. In the LID model, 53.65% of NAc shell GABAergic neurons presented an excitatory response to SMS, whereas 26.19% of control neurons presented an excitatory response, a proportion significantly greater than that of neurons showing an inhibitory or no response (Figure 4 and Appendix S4A–C). Single‐cell recordings revealed a significant increase in firing frequency and conditioned excitation of NAc shell neurons in LID rats within 0–100 s following SMS, aligning with the behavioral time window for CPP formation (Appendix S4D,E). The total discharge count of GABAergic neurons in the NAc shell of the LID + SMS group was significantly greater than that observed in the control group, providing direct evidence that SMS increases the excitability of these neurons (Figure 4 and Appendix S4C). Quantitative analysis via *Z* scores comparing neuronal activity 100 s before and after stimulation confirmed a significant increase in GABAergic neuron activity on the conditioned side following SMS (Figure 4G).

### 3.5 Inhibition of the GABAergic System in the NAc Shell Reverses the Effects of SMS in LID Rats

**FIGURE 5:**
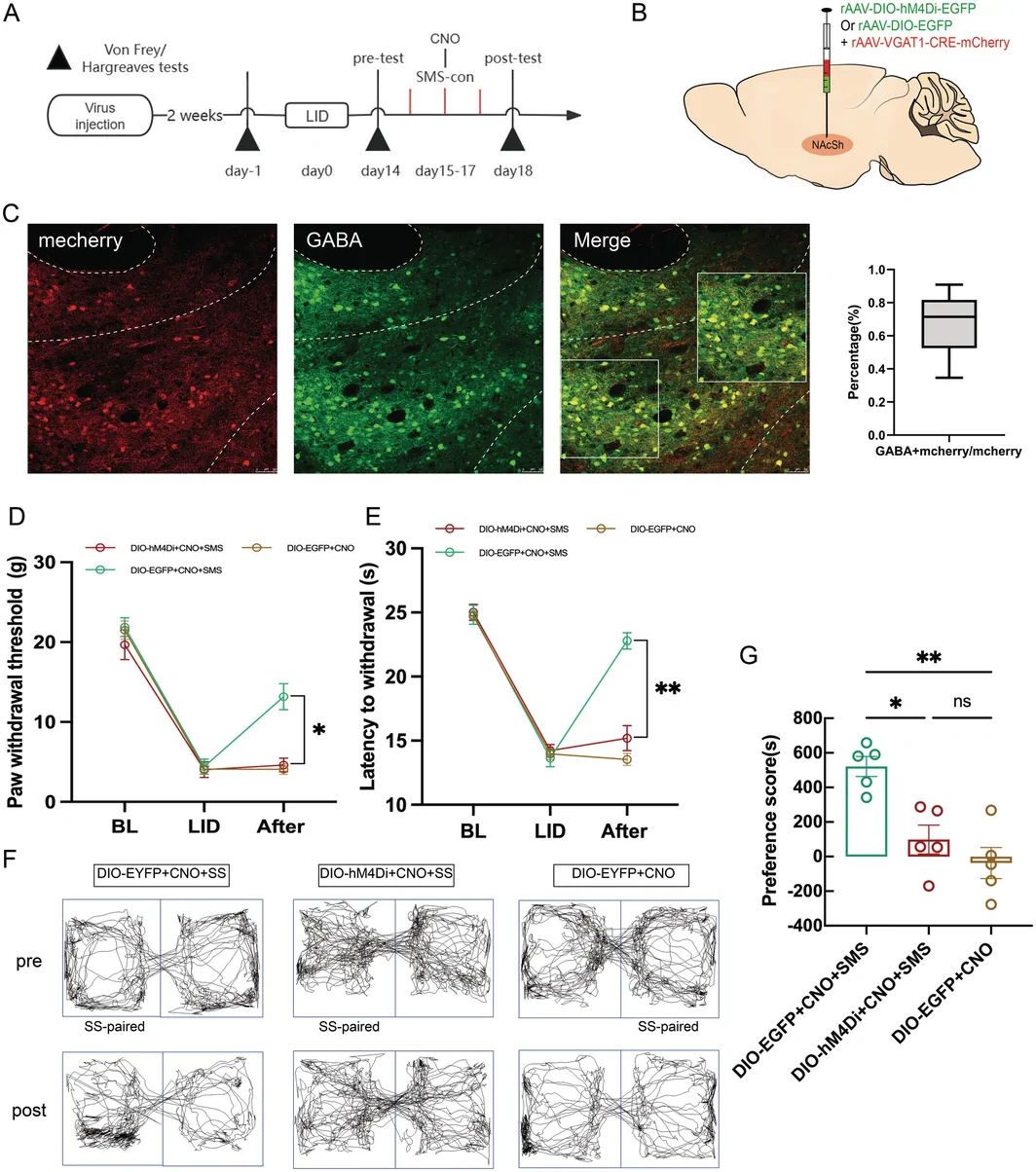
Inhibition of GABAergic neurons via chemical genetics in the NAc shell reverses the effects of SMS in LID rats. (A) Schematic showing the protocol of virus injection and the CPP experiment. (B) The injection of rAAV‐DIO‐hM4Di‐EGFP or rAAV‐DIO‐EGFP and rAAV‐VGAT1‐CRE‐mCherry into the NAc shell in rats. (C) Representative images of mCherry expression, GABA immunostaining, and colocalization in the NAc shell. (D) Effect of SMS after the intraperitoneal injection of clozapine‐N‐oxide (CNO) on the paw withdrawal threshold in LID rats. Two‐way RM ANOVA was used for data analysis; *F* (4, 24) = 5.956, DIO‐hM4D + CNO + SMS versus DIO‐EGFP+CNO + SMS (*p < 0.05). (E) Effect of SMS after intraperitoneal injection of clozapine‐N‐oxide (CNO) on the latency to withdrawal in LID rats (*n* = 5 in each group; two‐way RM ANOVA was used for data analysis; *F* (4, 24) = 33.31, DIO‐hM4D + CNO + SMS versus DIO‐EGFP+CNO + SMS **p < 0.01). (F) Representative activity trajectories of real‐time movement traces among the groups during the CPP test. (G) CPP scores of the animals in each group (*n* = 5 in each group; one‐way ANOVA was used for data analysis; *F* = 11.91, *p < 0.05, **p < 0.01).

A chemogenetic approach was used to specifically inhibit GABAergic neurons in the NAc shell. Chemogenetic suppression of these neurons reversed the analgesic effects of SMS. SMS‐induced increases in paw withdrawal threshold and latency to withdrawal were significantly diminished following the intraperitoneal injection of CNO in LID rats (Figure 5D,E). CNO pretreatment also attenuated SMS‐induced CPP (Figure 5).

Furthermore, the SMS‐induced improvements in paw withdrawal threshold and latency to withdrawal in LID rats were reversed by local administration of Bic, a GABA_𝐴_ receptor antagonist (Appendix S5B,C). The CPP scores were significantly lower in the LID + SMS + Bic group than in the LID + SMS + DMSO group (Appendix S5A–E), indicating that GABA_𝐴_ receptor inactivation in the NAc shell abolished the SMS‐induced increase in CPP.

### 3.6 SMS‐Induced Analgesia and CPP Involve the NAc Shell –IL Circuit

**FIGURE 6:**
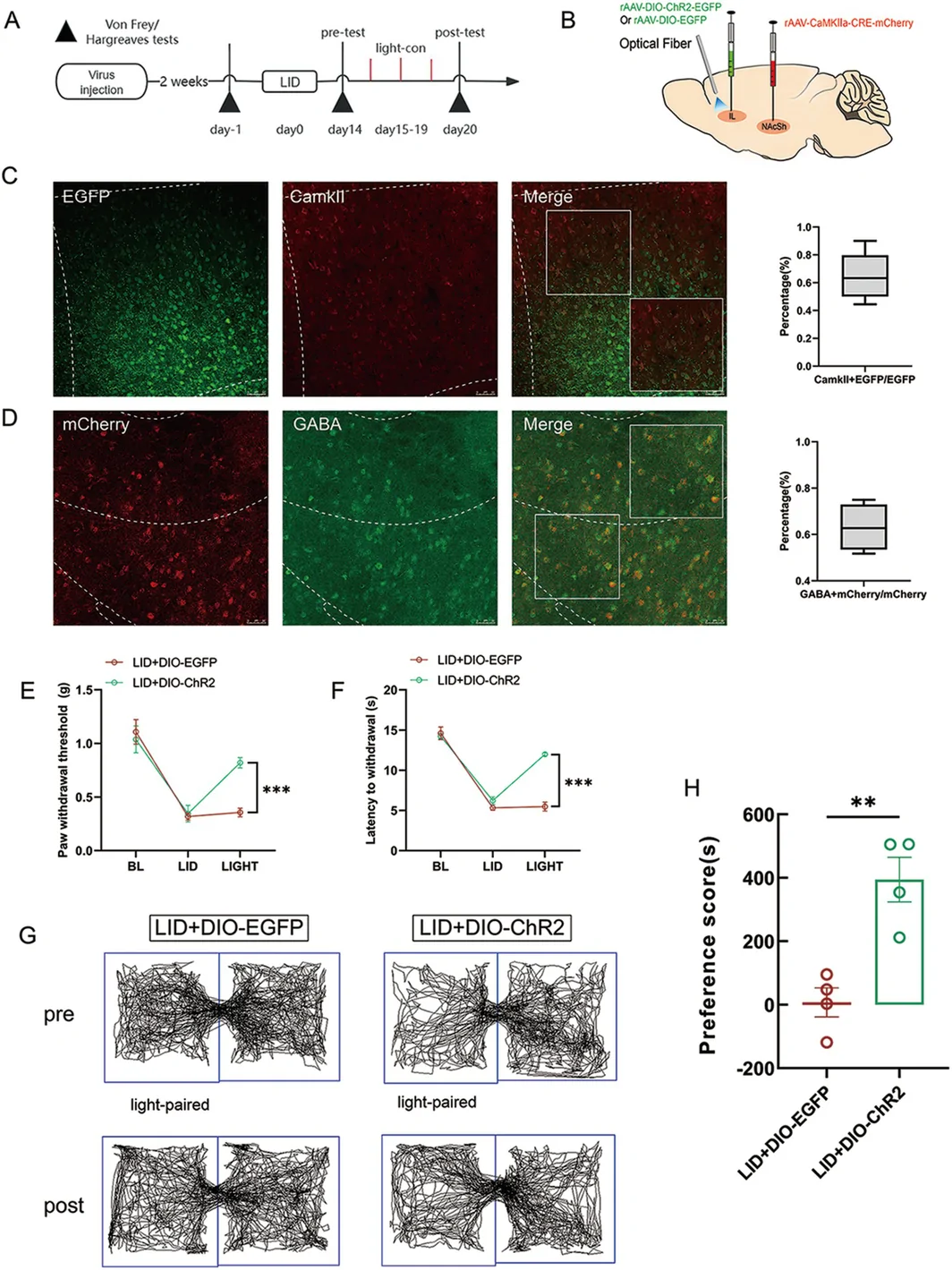
The IL‐to‐NAc Shell^GABA^ circuit is involved in SMS‐induced pain relief and affective motivational behaviors. (A) Schematic showing the protocol used for virus injection and the CPP experiment. Based on pilot data showing an effect size of 1.8 for paw withdrawal threshold, with α = 0.05 and power = 0.8, a minimum of 4 animals per group was required. (B) Injection of rAAV‐DIO‐ChR2‐EGFP or rAAV‐DIO‐EGFP into the bilateral IL, embedding of optical fibers and injection of rAAV‐CamKIIa‐CRE‐mCherry into the NAc shell in mice. (C) Representative images of EGFP expression, CaMkII immunostaining, and colocalization in the IL. (D) Representative images of mCherry expression, GABA immunostaining and colocalization in the NAc shell. (E) The effect of photoactivation on the paw withdrawal threshold in LID mice (*n* = 5 in the LID+DIO‐EGFP group, *n* = 4 in the LID+DIO‐ChR2 group; two‐way RM ANOVA was used for data analysis; *F* (2, 14) = 11.25, ***p < 0.001). (F) Effect of photoactivation on the paw‐withdrawal latency threshold in LID mice (*n* = 5 in the LID+DIO‐EGFP group, *n* = 4 in the LID+DIO‐ChR2 group; two‐way RM ANOVA was used for data analysis; *F* (2, 14) = 24.53, ***p < 0.001). (G) Representative activity trajectories of real‐time movement traces among the groups during the CPP test. (H) CPP scores of the animals in each group (*n* = 4 in each group; paired *t*‐test was used for data analysis; **p < 0.01).

The NAc receives input from the mPFC, processes pain signals, and integrates rewarding valence in the descending pathway [34, 35, 36]. Anatomical studies have shown that PL fibers are broadly distributed across the core and shell regions, whereas IL fibers project more selectively to the NAc shell [37, 38, 39]. To assess the involvement of the IL–NAc shell pathway in SMS‐induced pain relief and CPP, bilateral IL regions were injected with rAAV‐DIO‐ChR2‐EGFP (light‐sensitive channel) or a control virus (rAAV‐DIO‐EGFP), while the NAc shell received rAAV‐CaMKIIα‐CRE‐mCherry. Fiber optics were implanted to enable pathway‐specific light activation. The effects of optogenetic activation on nociceptive thresholds and affective behaviors were evaluated in conjunction with LID model induction and CPP testing (Figure 6A,B). Viral vector delivery enabled specific colocalization of IL^Glut^ neurons to NAc shell neurons, a subset of which are GABAergic (Figure 6C,D). Light activation of this pathway significantly increased the paw withdrawal threshold and latency to withdrawal and induced CPP in LID mice (Figure 6E–H).

A chemogenetic approach was employed to further validate the role of the IL‐NAc shell pathway in SMS‐induced analgesia. The IL of C57BL/6J mice was bilaterally injected with rAAV‐EF1a‐DIO‐hM4D(Gi)‐EGFP‐WPREs (hM4Di DREADDs), and the NAc shell was bilaterally injected with rAAV‐CaMKIIα‐CRE‐WPRE‐hGH PA (Appendix S6A,B). As anticipated, the SMS‐induced increase in paw withdrawal threshold and latency to withdrawal was abolished following intraperitoneal administration of the hM4Di agonist clozapine‐N‐oxide (CNO) (Appendix S6C,D). CNO injection significantly reduced SMS‐induced place preference in LID mice (Appendix S6E). The results suggest that the reversal of SMS‐induced CPP by inhibition of the NAc shell GABAergic system may reflect a disruption of the rewarding valence associated with pain relief, consistent with the established role of the NAc in integrating the affective state.

## 4 Discussion

SMS is a novel neuromodulatory approach that is different from traditional thermal or electrical methods and functions as a bioelectronic strategy to modulate peripheral‐to‐central neural signaling and induce rapid, opioid‐independent analgesia [40, 41]. The present study revealed that SMS exerts strong analgesic effects by activating the IL‐to‐NAc pathway, a key neural circuit involved in top‐down pain modulation. These findings identify the mechanistic basis of SMS‐induced analgesia and underscore the capacity of peripheral neuromodulation to engage central pain‐regulatory networks. Moreover, the results identify the subcutaneous layer as a viable therapeutic target for neuromodulatory interventions, presenting a framework with significant implications for clinical pain management. By addressing the limitations of pharmacological treatments, SMS offers a noninvasive, opioid‐sparing strategy with potential applications in chronic pain, postoperative recovery, and neuropathic disorders [39]. This work establishes a foundation for the use of subcutaneous neuromodulation to selectively engage top‐down neural pathways for pain relief.

In this study, validation in two LID and SNI animal models demonstrated that SMS effectively alleviates pain, which aligns with our clinical observations. Clinically, ankle acupuncture, a form of subcutaneous neuromodulation, was introduced in the 1970s [42, 43] and has been shown to have analgesic effects on various pathological pain conditions, including low back pain, cancer‐related pain, and postoperative recovery [44, 45, 46]. The strategic application of subcutaneous stimulation offers several clinical advantages: (1) a favorable safety profile due to the avascular nature of appendicular regions, which significantly lowers the risk of hemorrhage and infection; (2) minimally invasive delivery via percutaneous needle insertion, avoiding the need for surgical implantation; (3) operational simplicity, as it requires only subcutaneous needle placement rather than complex electrode positioning; and (4) superior patient tolerability, with no sustained adverse sensations such as paresthesia or pressure and discomfort limited to brief cutaneous penetration.

SMS displays analgesic efficacy comparable to traditional acupuncture, whereas tactile stimulation or nonpenetrating sham acupuncture does not elicit similar effects [47, 48, 49]. In the present study, the SMS operates independently of the opioid system, as its analgesic response remains largely unaffected by the opioid antagonist naloxone. Furthermore, SMS demonstrates a rapid onset of action, within 2 min, compared with the 10–30 min typically required for opioid analgesics [50, 51, 52]. Unlike opioids, SMS circumvents risks such as addiction and respiratory depression, presenting a safer modality for pain management. This is particularly relevant for chronic pain conditions, where prolonged opioid use is associated with dependence and numerous adverse outcomes. SMS thus represents a promising alternative for long‐term pain treatment.

The CPP is a well‐established behavioral paradigm in preclinical pain research used to evaluate the affective component of pain and the rewarding aspects of pain relief [53, 54]. In the present study, SMS‐induced analgesia elicited CPP in two animal pain models, indicating that SMS produces measurable changes in affective behavior. These findings provide insight into the affective and motivational responses associated with SMS treatment, advancing the understanding of its analgesic mechanisms. SMS induces analgesia and elicits affective and motivational changes following pain relief. The CPP was observed in pain models but not in sham‐operated controls, indicating that SMS elicits motivational responses specifically in pain. This suggests that SMS does not possess inherent rewarding properties but facilitates negative reinforcement by alleviating an aversive state, which is consistent with established theories of pain relief‐induced motivation.

Contralateral SMS also significantly attenuated nociceptive hypersensitivity in both the LID and SNI models, suggesting the involvement of more brain regions in mediating the therapeutic effects of SMS. Previous studies have shown that GABA is key for inhibiting pain transmission along specific neural pathways, supporting its contribution to descending pain modulation [55, 56]. A recent study demonstrated that GABA_𝐴_ receptors regulate neural activity within the NAc [57]. Exogenous GABA administration was shown to suppress the activity of pain‐excited neurons via GABA_𝐴_ receptor modulation in the NAc, resulting in analgesia. In this study, SMS selectively activated the GABAergic system in the NAc shell, and this activation appears to be critical for both its analgesic effects and the regulation of pain‐related affective behaviors. Inhibition of GABAergic neurons or GABA_𝐴_ receptors in the NAc shell reversed SMS‐induced analgesia and CPP, confirming the critical mediating role of these neurons in the effects of SMS. Further analyses revealed the involvement of the neural pathway from IL glutamatergic neurons to NAc shell GABAergic neurons in SMS‐induced pain relief and affective behavioral modulation. Optogenetic activation of this pathway significantly elevates pain thresholds and induces place preference. These findings elucidate the role of IL–NAc shell top–down circuits in mediating the effects of SMS and provide mechanistic insight into how peripheral neuromodulation engages central pathways to achieve analgesia. Notably, inhibition of the NAc may simultaneously affect reward processing, and therefore, the possibility of direct reward interference on CPP cannot be entirely ruled out. Future studies could further validate these findings using more direct behavioral or neurophysiological measures.

The present study has several limitations that should be acknowledged. First, although Piezo channels were identified as essential mediators of SMS‐induced mechanical analgesia, the specific subtype(s) involved, such as Piezo1 or Piezo2 [26, 58], remain to be conclusively determined. Second, although SMS elicits rapid analgesic effects, the underlying mechanisms remain incompletely understood. The possibility that nonmechanical factors, such as chemical or biochemical mediators, contribute to this effect warrants further investigation. Third, the signal transduction mechanisms by which SMS‐generated mechanical stimuli are converted into electrical signals and transmitted to spinal and supraspinal structures remain poorly defined, limiting a comprehensive understanding of the neuromodulatory pathway [26]. Fourth, the inclusion of both sexes and the use of appropriate statistical models (e.g., a two‐way ANOVA with factors for sex and treatment) are essential to ensure generalizability [59]. Finally, the tactile stimulation produced as the needle penetrates the skin elicited the transient hindlimb withdrawal response. In addition, in clinical practice, patients may perceive a mild, non‐painful distending sensation during needle retention, which is distinct from the sharp pain. Hence, we cannot entirely rule out the possibility that the reduction in nociceptive/pain‐related behaviors may result from a DNIC/CPM‐like effect (“pain inhibits pain”) [60]. However, a DNIC/CPM‐like mechanism alone does not fully account for the observed effects. (1) DNIC/CPM is fundamentally based on the principle that a distant, sustained, or tonic noxious stimulus (conditioning stimulus) can suppress the perception of a noxious test stimulus applied to a different body region [61]. In contrast, the sensation induced by SMS is very mild and qualitatively different from that of transcutaneous electrical nerve stimulation or acupuncture. During SMS, patients typically do not experience a sustained “noxious” level of stimulation. (2) Our results indicate that SMS engages central pain‐suppression pathways for analgesia, which are anatomically and functionally distinct from the spinal–brainstem–spinal feedback loops that underlie DNIC/CPM [60]. (3) A key feature of DNIC/CPM is that its effects are tightly linked to the presence of the conditioning stimulus, usually subsiding rapidly after its termination [61]. SMS, however, produces pronounced aftereffects. Addressing these considerations in future studies may facilitate the development of more precise and effective SMS‐based analgesic interventions. These results highlight the necessity for validation studies incorporating advanced neuroimaging and genetic methodologies to delineate the dynamic modulation of neural circuits and brain networks by SMS. Such approaches are essential for establishing a rigorous mechanistic framework to support the clinical translation of SMS.

## Funding

This work was supported by Shanghai Key Laboratory of acupuncture mechanism and acupoint function, 21DZ2271800. New Quality Clinical Specialty Program of High‐end Medical Disciplinary Construction in Shanghai Pudong New Area, 2025‐PWXZ‐17. Shanghai Natural Science Foundation, 24ZR1466300. Shanghai Pudong New Area Health Commission, PDZY‐2025‐0707, PDZY‐2026‐1204.

## Ethics Statement

All experimental procedures were performed according to the National Institutes of Health (NIH) guidelines for the care and use of laboratory animals and were approved by the Animal Ethics Committee of Shanghai University of TCM (Approval No. SZY201710008; Animal License No. PZSHUTCM2212090004).

## Conflicts of Interest

The authors declare no conflicts of interest.

## Supporting information


**Video S1:** Demonstrates the administration of SMS and the subsequent behavioral responses in SNI rats.

**Video S2:** Demonstrates the administration of sham SMS and the subsequent behavioral responses in SNI rats.

**Video S3:** Illustrates the spontaneous activity of SNI rats after receiving SMS.

**Appendix S1:** Characterizing the role of SMS in neuropathic pain through stimulus location.
**Appendix S2:** SMS induced analgesia in female rats with SNI.
**Appendix S3:** Behavioral changes in rats between SMS and SHAM SMS groups.
**Appendix S4:** Specific activating effects of SMS on NAc Shell GABAergic neurons revealed at the single‐cell level.
**Appendix S5:** GABA receptor antagonist in NAc Shell reverses the effects of SMS in LID rats.
**Appendix S6:** Inactivating IL–NAc Shell pathway reserves SMS‐induced analgesia and CPP behaviors.

## Data Availability

All the data needed to evaluate the conclusions in the paper are presented in the paper and/or the Supporting Information. The datasets used and/or analyzed during the current study are available from the corresponding author upon reasonable request.
