# Supplementary material for: Subcutaneous Neuromodulation Activates Central Pain‐Suppression Pathways for Analgesia
Source: CNS Neurosci Ther. 2026 Aug 27;32(8):e71004. doi: 10.1002/cns.71004 (PMC13522390; doi:10.1002/cns.71004)
Supplement: Supplementary file 1 [file CNS-32-e71004-s001.docx]

**Appendix Materials for**

**Subcutaneous neuromodulation activates central pain-suppression pathways for analgesia**

**Qiuyong Li et al.**

**Corresponding author: Sheng Liu, liusheng@shutcm.edu.cn**

**This PDF file includes:**

**Appendix Materials and Methods**

**Appendix S1 to S6**

**Video S1-S3**

**Materials and Methods**

**Animals**

8 to 10-week-old adult Sprague-Dawley rats (250–300 g; SLAC) and Male C57BL/6J 520 (6-8 weeks old; SLAC) were used in this study. Animals were purchased from the Laboratory Animal Center of Shanghai University of Traditional Chinese Medicine. The experiments were conducted in accordance with NIH standard guidelines for the use and care of experimental animals. These rats were housed, 3–5 per cage in a colony, in a stable environment (23– 25°C ambient temperature) with ad libitum access to standard lab mouse pellet food and water on a 12 h light/12 h dark cycle (lights on from 07:00 to 19:00). All experiments were conducted with the approval of the Shanghai University of TCM Animal Ethics Committee (SZY201710008). The animal license was No. PZSHUTCM2212090004.

**Reagent and drug**

Zymosan (Sigma-Aldrich, Z4250) was dissolved in saline to a concentration of 20 mg/ml. Naloxone hydrochloride (MedChem Express, HY-17417), an opioid receptor antagonist, was administered intraperitoneally at a dose of 4 mg/kg. The following reagents were also employed: bicuculline (MedChemExpress, HY-N0219), and primary antibodies against GABA (Abcam, ab17413), c-Fos (Abcam, ab208942), and CaMKⅡ (Abcam, ab52476).

For retrograde tracing, optogenetic, and chemogenetic manipulation, the following adeno-associated viruses were used: rAAV-hSyn-DIO-hChR2(H134R)-EGFP-WPRE-hGH polyA (2.0×10¹²vg/mL), rAAV-CaMKⅡa-CRE-WPRE-hGH PA (5×10¹²vg/mL), rAAV-EF1a-DIO-hM4D(Gi)-EGFP-WPREs (5×10¹² vg/mL), rAAV-EF1a-DIO-EGFP-WPRE-hGH polyA (5.6×10¹² vg/mL), and rAAV-VGAT1-CRE-mCherry-WPRE-hGH polyA (3.6×10¹² vg/mL). All viral constructs were obtained from Brain VTA. GsMTx4 (10 µM in saline; Abcam, ab141871) was used to selectively inhibit Piezo channels. SKF 96365 (50 µM in DMSO; MedChemExpress, HY-13248), a nonselective TRP channel blocker, was applied to inhibit transient receptor potential (TRP) channels. Both compounds were delivered via local subcutaneous injection (10 µL) at the SMS site 30 minutes before stimulation. Control animals received equivalent volumes of saline or DMSO as vehicle.

**Spared Nerve Injury (SNI)**

As our detailed previously ^1^: Surgical procedures were conducted under 5% isoflurane anesthesia. A longitudinal incision was made along the lateral aspect of the right femur, followed by blunt dissection of the biceps femoris muscle to expose the sciatic nerve trifurcation. Distal to the sciatic trifurcation, the common peroneal and tibial nerve branches were isolated, double-ligated with 6-0 surgical silk sutures, and excised to remove 2 mm nerve segments, while the sural nerve was preserved intact.

**Localized DRG Inflammation (LID)**

As described previously ^2^, inflammatory radiculopathy was induced by administering zymosan A to the dorsal root ganglia (DRG). Under isoflurane anesthesia, a 0.5-inch 30-gauge needle was inserted perpendicularly into the L4 (for mice) or L5 (for rats) intervertebral foramen. A sterile solution of zymosan A (2 mg/ml in saline, 10 μl) was then injected to induce localized DRG inflammation. Sham control animals underwent the same surgical procedure, including DRG exposure, but did not receive any injection.

**Subcutaneous mechanical stimulation (SMS)**

To accommodate the anatomical dimensions of the animals, a stainless-steel needle (0.08 mm diameter × 5 mm length) was fabricated by modifying a standard dispensing needle (0.08 mm diameter × 15 mm length). The needle was cut to the required length, with its proximal end curled into a loop and the distal tip ground to a blunt point using a precision needle grinder. Prior to use, the needle was sterilized by immersion in hydrogen peroxide solution ^3^.

For subcutaneous stimulation in both the LID model and the distal segment of the SNI model, animals were gently restrained following standard protocols. After disinfecting the skin with iodine and sterilizing the needle, the implantation procedure was performed as follows: The needle was inserted at a location 5 mm lateral to the achilles tendon and 7 mm above the ankle joint (3 mm for mice), using needle-holding forceps for precise manipulation. The initial insertion was performed at a 30° angle relative to the skin surface, followed by advancement parallel to the dermal plane. The implanted needle was then secured with medical tape. In sham control groups, needles were placed adjacent to but not penetrating the skin, with identical tape fixation to maintain procedural consistency. For stimulation of the proximal segment in SNI-injured nerves, the needle was implanted into the anatomical depression along the midline of the tibia near the patellar region. The needle was left in place for 20 min.

**Mechanical Allodynia Assessment (von Frey Test)**

Subjects underwent a 3-day environmental acclimation period in testing chambers maintained at 22±1°C with a 12-hour light/dark cycle. Daily habituation involved placing animals in transparent polycarbonate enclosures (20×15×10 cm) positioned on elevated wire mesh platforms for 30 minutes to minimize handling stress. Calibrated von Frey monofilaments (0.008–300 g force range, North Coast Medical) were applied perpendicularly to the plantar surface of the hindpaw using an ascending stimulus paradigm (starting with the 2 g filament), following established protocols for neuropathic pain assessment (Dixon, 1980). A positive response was defined as rapid paw withdrawal, sustained paw elevation (>1 second), or aversive grooming behavior. Testing ceased after five consecutive consistent responses following the initial reaction ^4^. The 50% mechanical withdrawal threshold was calculated using Dixon’s up-down method (modified Spearman-Kärber). An inter-stimulus interval of 5 minutes was enforced between trials to prevent stimulus-induced sensitization.

**Thermal Hyperalgesia Evaluation (Hargreaves Test)**

A radiant heat apparatus (Model 390G, IITC Life Science) was used, with the heat beam directed through a 3-mm tempered glass platform. The system was calibrated to deliver 15% of maximal intensity, resulting in a surface temperature of approximately 50°C at the target site. The heat beam was focused on the central plantar region of the affected hindpaw. Each subject underwent three trials with 10-minute inter-trial intervals to avoid thermal tissue damage. Cutoff latencies were set at 30 seconds for rats and 20 seconds for mice. Paw withdrawal latency was automatically recorded via an integrated photodetector system. Mean latency values were calculated from triplicate measurements, with coefficient of variation <10% considered valid for statistical analysis ^5^.

**Conditional place preference (CPP)**

CPP was performed as our detailed previously ^6^. A standard two-chamber balanced design was used. On the preconditioning day, animals were allowed to travel the chambers freely for 15 min, and the amount of time spent in each chamber was recorded. Rats that spent > 720 s of the total time (900 s) on one side were eliminated from subsequent procedures. In fact, none of the animals had a substantial initial bias for either compartment in our experiment. Next, two conditioning sessions (for SMS and gentle handling) were performed for three consecutive days. Animals were treated with either SMS or gentle handling for 30 min each in the morning and afternoon. We immediately placed the animals into the pairing chamber and confined them to the chamber for 30 min after SMS administration or gentle handling. For every conditioned animal, SMS and gentle handling were carried out alternatively in the morning and afternoon sessions. The morning and afternoon SMS stimulation and gentle handling were conducted at least 4 h apart. A preference test was conducted 3 days after conditioning. Animals were placed in the CPP apparatus with a door opened in the middle for 15 min to allow access to the apparatus’s whole parts in this phase. The preference scores were analyzed by calculating the amount of time the animals spent in the SMS paired chamber minus the time they spent in the gentle handling chamber.

**Locomotor Activity**

Rats were randomly assigned to either the SMS group or the SHAM SMS group (n = 5 per group). Spontaneous locomotor activity was assessed using a black acrylic open arena (100 cm × 100 cm × 40 cm) equipped with an infrared video tracking system (EthoVision XT 14, Noldus Information Technology, Netherlands) ^18^. Behavioral testing was conducted 15 minutes after the intervention in a quiet, dimly lit environment at 22 ± 1 °C. Prior to any intervention or testing, the animals were acclimated to the testing room for 60 minutes. The testing arena was cleaned with 75% ethanol between trials. The total distance traveled was automatically recorded and analyzed by the system.

**Assessment of pain-related behaviors (limping, licking and grooming behaviors)**

Under constant environmental conditions, rats were placed individually in a transparent acrylic test box (20 cm × 15 cm × 18 cm) each day to observe spontaneous pain-related behaviors ^19^. Behavioral recordings began 15 minutes after the intervention and lasted for 20 minutes. Prior to the intervention, rats were acclimated to the chamber for 30 minutes. Grooming behavior was defined as stereotyped movements, including paw licking, fur grooming, and body rubbing. All tests were conducted by the same operator to minimize systematic error. The total numbers of spontaneous paw withdrawals, paw licks, and grooming episodes were quantified over the 20-minute observation period.

**Scanning Electron Microscopy (SEM)**

To confirm the successful anatomical targeting of SMS needles in animal models, a localization assay was performed ^7^. Briefly, rats were allowed to ambulate with implanted needles for 5 min before anesthesia with sodium pentobarbital (50 mg/kg, i.p.). The cardiovascular system was perfused transcardially with 0.9% physiological saline for 15 min, followed by regional perfusion of the needle-implanted hindlimb with 2% glutaraldehyde fixative for 12 h to preserve tissue morphology. Using forceps, the fixed hindlimb tissue was excised and placed under a dissecting microscope (Leica M165FC). A sterile disposable blade was used to make longitudinal incisions parallel to the needle shaft and perpendicular to the skin surface, starting 5 mm from the needle insertion site. Additional orthogonal cuts were made to isolate a rectangular tissue block (~10 × 10 × 5 mm) containing the needle tract. The tissue block was processed as follows: (1) Washing: Twice with 0.1 M sodium cacodylate buffer (pH 7.4) for 10 min each; (2) Post-fixation: Immersion in 1% osmium tetroxide for 2 hours at 4°C; (3) Dehydration: Graded ethanol series (30%, 50%, 70%, 90%, 100% v/v), 15 min each; (4) Intermediate Solvent: Infiltration with acetone for 20 min. After critical point drying (CPD030, Leica) to prevent tissue distortion, the specimen was mounted on an aluminum stub with double-sided carbon tape, exposing the needle tract and adjacent tissue. The stub was coated with Au-Pd (5 nm thickness) using an ion sputter coater (Leica EM ACE600). Imaging was performed using a Quanta FEG 250 environmental SEM (Thermo Fisher Scientific) at 10 kV, with secondary electron detection for high-resolution visualization of needle-tissue interfaces ^8^.

**Immunofluorescence staining**

Rats or mice were deeply anesthetized using pentobarbital sodium (100 mg/kg, ip), sequentially perfused transcardially with saline (200 mL for rats, 30 mL for mice) and 4% paraformaldehyde (PFA) in 0.1 mol/L phosphate buffer (PB) (250 mL for rats, 30 mL for mice), and brains were collected from each animal, fixed overnight in 4% PFA, and transferred to 30% sucrose for 5-7 days until the tissue was saturated and sank to the bottom of the solution. Samples were then cut to yield a series of 30 μm-thick coronal sections using a chilled (−25°C) cryostat instrument, with the resultant sections being transferred to PBS.

The staining of these sections was conducted as in our prior study ^9^. Briefly, sections were washed thrice with PBS, blocked for 2 h with 1% BSA at 4°C, and probed for 48 h with the primary antibodies at 4°C. Following three subsequent washed with PBS, sections were stained for 2 h with corresponding fluorescent secondary antibody (1:500). Sections were then mounted on adhesive slides to which coverslips and fluorescence decay-resistant medium were applied. A Leica Laser Scanning Confocal Microscope was then used to analyze these stained sections. The best standard stereotaxic plane sections of the IL and the NAc shell were identified as per Paxinos and Watson's atlas ^10^, with numbers of positive cells per section then being assessed (20 x). An automatically generated 250×600 μm rectangle was used to denote the IL and the NAc shell in each section, and an analytical software was used to calculate the number of stained nuclei per section. Numbers of positive nuclei per section were thereby determined and averaged to yield representative results for analysis. For each rat, Fos⁺/GABA⁺ neurons were counted across 3–4 coronal sections of the NAc, and the average percentage per animal was used for statistical analysis.

**In vivo electrophysiological recordings**

Adult male Sprague-Dawley rats (270–300 g pre-surgery weight) were anesthetized with intraperitoneal sodium pentobarbital (induction: 50 mg/kg; maintenance: 15 mg/kg/h) and secured in a stereotaxic frame (David Kopf Instruments). Body temperature was maintained at 37 ± 0.5°C using a feedback-controlled heating pad. After scalp incision and skull exposure, bilateral craniotomies were drilled at the following coordinates relative to bregma for nucleus accumbens (NAc) shell implantation: anteroposterior (AP) +1.5 mm, mediolateral (ML) ±1.0 mm, dorsoventral (DV) -6.5 mm (Paxinos & Watson, 2014). A 16-channel microwire array (50 μm diameter, FHC Inc.) was lowered into the NAc at a rate of 0.1 mm/min using a microdrive system. The array was secured to the skull with dental cement and stainless-steel anchor screws. Postoperatively, rats were single-housed and administered penicillin G (160,000 IU/day, intramuscular injection) for 5 consecutive days to prevent infection.

Extracellular recordings were conducted using a Cerebus high-density neural recording system (Blackrock Neurotech, Salt Lake City, UT, USA). Animals were connected via a headstage interface to a multichannel amplifier (gain: 500×, bandpass filter: 100 Hz–8 kHz). Signals were digitized at 40 kHz, high-pass filtered at 250 Hz, and stored on a dedicated workstation. A commutator system allowed unrestrained movement during recording sessions. Raw data were processed offline using Offline Sorter v2.8 (Blackrock Neurotech). SMS was applied over a 10-second window at time 0, with electrophysiological data aligned to this event. Baseline activity was recorded for 100 seconds prior to SMS, and post-SMS activity was analyzed in 100-second bins thereafter.

Spike sorting and unit inclusion criteria: Single units were isolated using Offline Sorter (Plexon) based on principal component analysis and waveform parameters. Only units with a clear refractory period (>1 ms) and stable waveform throughout the recording were included. Among these, putative GABAergic interneurons were identified as those with high firing rate (>15 Hz) and narrow spike width (<225 μs). Neuronal activity was analyzed using NeuroExplorer (Nex Technologies) and MATLAB (MathWorks). Firing rates were Z-score normalized to baseline (mean ± SD). Responsive neurons were defined as those showing a significant change in firing rate post-SMS relative to baseline (Wilcoxon signed-rank test, P < 0.05). Inter-group differences in baseline firing rates were assessed using the Wilcoxon rank-sum test. Cross-correlogram analysis (bin width: 1 ms) was performed to exclude multi-channel recordings of the same neuron; units showing >5% peak correlation was excluded from parallel channel analyses ^11^.

**Cannula surgeries and microinjection**

Rats were anesthetized with intraperitoneal sodium pentobarbital (induction: 50 mg/kg; maintenance: 15 mg/kg/h, i.p.) and positioned in a stereotaxic frame (David Kopf Instruments). A guide cannula (62001, RWD Life Science, Shenzhen, China) was implanted into the nucleus accumbens (NAc) shell at the following coordinates (relative to bregma, Paxinos & Watson, 2014): anteroposterior (AP) +1.5 mm, mediolateral (ML) ±1.1 mm, dorsoventral (DV) -6.5 mm. The cannula was secured to the skull with stainless-steel anchor screws and dental acrylic cement, with obturators immediately inserted to maintain sterility and ensure the tips reached the targeted injection site ^12^.

Thirty minutes prior to SMS, rats underwent bilateral microinjection into the NAc shell. Obturators were removed from the guide cannula, and a 33-gauge injector needle (42–0210, Hamilton, Bonaduz, Switzerland) was advanced such that its tip extended 2 mm beyond the cannula terminus. Animals received either: Bicuculline (Bic): 3.0 nmol/0.3 μl (10 mM in DMSO, MCE, HY-N0219, Monmouth Junction, NJ, USA). Vehicle control: 0.3 μl DMSO (Sigma-Aldrich, D2650). Solutions were infused at a rate of 0.3 μl/min using a microinfusion pump (CMA 100, Carnegie Medicine), with the injector needle remaining in place for an additional 3 minutes post-infusion to facilitate diffusion .

**Viral Injection**

For retrograde tracing, rAAV-VGAT1-CRE-mCherry-WPRE (3.6×10¹² vg/mL) was injected bilaterally at 20 nL/min to bilateral NAc shell ( AP +0.5 mm, ML ±1.1 mm, DV -4.5 mm). For optogenetic stimulation, 80 nL of rAAV-hSyn-DIO-hChR2(H134R)-EGFP -WPRE -hGH (2.0×10¹² vg/mL) or rAAV-EF1α-DIO-EGFP-WPRE-hGH (5.6×10¹² vg/mL) was injected at 20 nL/min to right IL (AP +1.6 mm, ML -0.25 mm, DV -2.8 mm) or 120 nL of rAAV-CaMKIIα-CRE-WPRE-hGH (5×10¹² vg/mL) was injected at 20 nL/min to right NAc shell (AP +0.5 mm, ML +1.1 mm, DV -4.5 mm).

For chemogenetical experiment, a 1:1 mixture (total 120 nL) of rAAV-EF1α-DIO-hM4D(Gi)-EGFP-WPRE (5×10¹² vg/mL) and rAAV-VGAT1-CRE-mCherry-WPRE (3.6×10¹² vg/mL) was injected bilaterally at 20 nL/min to bilateral NAc shell ( AP +0.5 mm, ML ±1.1 mm, DV -4.5 mm). Clozapine-N-oxide (CNO, 1 mg/kg) was dissolved in saline (1.0 mg/mL) and administered intraperitoneally 30 minutes prior to SMS ^13^.

**Optic Fiber Implantation**

Immediately following IL virus injection, a 200-μm diameter optic fiber (NA 0.37, Doric Lenses) was implanted to IL at coordinates: AP +1.6 mm, ML -0.25 mm, DV -2.5 mm (0.3 mm above injection site). The skull surface was sealed with Vetbond tissue adhesive (3M), and dental acrylic cement was applied to secure the fiber and cover exposed bone. Animals were recovered on a heated pad before returning to housing. Viral expression was allowed for 3 weeks prior to subsequent experiments ^14,15^.

**Optogenetic manipulation**

Implantable optic fibers (200 μm diameter, 0.37 NA, Doric Lenses) were connected to a 473 nm blue laser system (Cube 473-100, Cobolt) via optical fiber couplers (Doric Lenses). Stimulation parameters were controlled by a Doric NeuroStar interface: 6 mW power, 10 ms pulse width, 10 Hz frequency, delivered continuously for 30 minutes. In control groups, the same fiber-coupled laser system was used, but light emission was disabled to maintain procedural consistency. Optogenetic manipulations were performed during the conditioning phase of the conditioned place preference (CPP) paradigm. Mice with histologically verified viral injection misses or optic fiber misplacement (determined by post-hoc cresyl violet staining) were excluded from analyses ^16^.

**Statistical analysis**

Statistical graphs were generated using GraphPad Prism 8.0 (GraphPad Software) and TBtools ^17^. Experimental data are presented as mean ±SEM. For comparisons between two groups, paired or unpaired Student’s t-tests were used, depending on experimental design. For analyses involving three or more groups: non-normally distributed data: Friedman test followed by Dunn’s post-hoc test, normally distributed data: One-way ANOVA or two-way repeated measures ANOVA, followed by post hoc comparisons using Tukey’s test (for one-way ANOVA) or Bonferroni (for two-way repeated-measures ANOVA). Missing data were handled using mixed-effects ANOVA to account for repeated measures and inter-individual variability. Statistical significance was set at P < 0.05, with exact P values reported for all comparisons.

**Reference**

1.	Decosterd I, Woolf CJ. Spared Nerve Injury: An Animal Model of Persistent Peripheral Neuropathic Pain. *Pain.* 2000;87(2):149–158.

2.	Zhang L, Xie W, Zhang J, et al. Key Role of CCR2-expressing Macrophages in a Mouse model of Low Back Pain And Radiculopathy. *Brain Behav Immun.* 2021;91:556–567.

3.	Vera-Portocarrero LP, Cordero T, Billstrom T, et al. Differential Effects of Subcutaneous Electrical Stimulation (SQS) and Transcutaneous Electrical Nerve Stimulation (TENS) in Rodent Models of Chronic Neuropathic or Inflammatory Pain. *Neuromodulation.* 2013;16(4):328–335.

4.	Pitcher GM, Ritchie J, Henry JL. Paw Withdrawal Threshold in the Von Frey Hair Test is Influenced by the Surface on which the Rat Stands. *J Neurosci Methods.* 1999;87(2):185–193.

5.	Hargreaves K, Dubner R, Brown F, et al. A New and Sensitive Method for Measuring Thermal Nociception in Cutaneous Hyperalgesia. *Pain.* 1988;32(1):77–88.

6.	Tzschentke TM. Measuring Reward with the Conditioned Place Preference (CPP) Paradigm: Update of the Last Decade. *Addict Biol.* 2007;12(3-4):227–462.

7.	Abolfazl Mehdizadeh Kashi, Kobra Tahermanesh, Shahla Chaichian, et al. How to prepare biological samples and live tissues for scanning electron microscopy (SEM). *Galen Medical Journal.* 2014;3(2):63–80.

8.	Sun C, Muller E, Meffert M, et al. On the progress of scanning transmission electron microscopy (STEM) imaging in a scanning electron microscope. *Microsc Microanal.* 2018;24(2):99–106.

9.	Im K, Mareninov S, Diaz MFP, et al. An Introduction to Performing Immunofluorescence Staining. *Methods Mol Biol.* 2019;1897:299–311.

10.	Liang S, Wu S, Huang Q, et al. Rat Brain Digital Stereotaxic white Matter Atlas with Fine Tract Delineation in Paxinos Space and its Automated Applications in DTI Data Analysis. *Magn Reson Imaging.* 2017;43:122–128.

11.	Jeggo R, Zhao FY, Spanswick D. Electrophysiological techniques for studying synaptic activity in vivo. *Curr Protoc Pharmacol.* 2014;64:11.11.11–17.

12.	Hernández-López F, Rodríguez-Landa JF, Puga-Olguín A, et al. Analysis of Activity and Motor Coordination in Rats Undergoing Stereotactic Surgery and Implantation of a Cannula into the Dorsal Hippocampus. *Neurologia.* 2017;32(9):579–586.

13.	Pawela C, DeYoe E, Pashaie R. Intracranial Injection of an Optogenetics Viral Vector Followed by Optical Cannula Implantation for Neural Stimulation in Rat Brain Cortex. *Methods Mol Biol.* 2016;1408:227–241.

14.	Ung K, Arenkiel BR. Fiber-optic Implantation for Chronic Optogenetic Stimulation of Brain Tissue. *J Vis Exp.* 2012(68):E500–E504.

15.	Pochechuev MS, Bilan DS, Fedotov IV, et al. Real-time Fiber-optic Recording of Acute-ischemic-stroke Signatures. *J Biophotonics.* 2022;15(10):e202200050.

16.	Berglund K, Tung JK, Higashikubo B, et al. Combined Optogenetic and Chemogenetic Control of Neurons. *Methods Mol Biol.* 2016;1408:207–225.

17.	Chen C, Chen H, Zhang Y, et al. TBtools: An Integrative Toolkit Developed for Interactive Analyses of Big Biological Data. *Mol Plant.* 2020;13(8):1194–1202.

18.	Burdge J, Fried NT, Abdus-Saboor I. Using high-speed videography for objective and reproducible pain measurement on a mouse pain scale. *STAR Protoc.* 2021;2:100322.

19.	Draxler P, Moen A, Galek K, et al. Spontaneous, Voluntary, and Affective Behaviours in Rat Models of Pathological Pain. *Front Pain Res* 2021;2:672711.

**Appendix S1. Characterising the role of SMS in neuropathic pain through stimulus location**

**specificity and mechanistic intervention**

(A)Paw withdrawal threshold of SMS stimulation which performed at the lower end of SNI injured nerve (n = 6 in SNI+SHAM SMS group, n = 9 in SNI+SMS group; two-way RM ANOVA was used for data analysis; F (5, 65) = 4.110, 0h, SNI+SMS vs. SNI+SHAM SMS ***P* <0.01; 2h, SNI+SMS vs. SNI+SHAM SMS **P* <0.05, 4h, SNI+SMS vs. SNI+SHAM SMS **P* <0.05). (B) Latency to withdrawal of SMS stimulation which performed at the lower end of SNI injured nerve (n = 5 in SNI+SHAM SMS group, n = 6 in SNI+SMS group; two-way RM ANOVA was used for data analysis; F (5, 45) = 8.246, 0h, SNI+SMS vs. SNI+SHAM SMS ***P* <0.01; 2h, SNI+SMS vs. SNI+SHAM SMS ***P* <0.01; 4h, SNI+SMS vs. SNI+SHAM SMS **P* <0.05). (C) Paw withdrawal threshold of SMS stimulation which performed at the upper end of SNI injured nerve (n = 6 in each group.). (D) Latency to withdrawal of SMS stimulation which performed at the upper end of SNI injured nerve (n = 6 in each group). (E) Effect of SMS on the paw withdrawal threshold in SNI rats after naloxone intraperitoneal injection (n = 9 in the SNI+SMS group, n = 8 in the SNI+NX+SMS group; two-way RM ANOVA was used for data analysis; F (4, 46) = 11.34, SNI +SMS vs. SNI+NX+SMS *P* >0.05). (F) Effect of SMS on latency to withdrawal in SNI rats after naloxone intraperitoneal injection (n =9 in the SNI+SMS group, n = 8 in the SNI+NX+SMS group; two-way RM ANOVA was used for data analysis; F (4, 40) = 5.185, SNI+ SMS vs. SNI+NX+SMS *P* > 0.05). (G) Representative activity trajectories of Real-time movement traces among the groups during CPP test. (H) CPP scores of animals in each group (n =6 in SNI+SMS group, n =8 in SNI+NX+SMS group, n =9 in SNI+LIDO+SMS group; one-way ANOVA was used for data analysis; F = 12.65, SNI+SMS vs. SNI +LIDO+SMS ***P* <0.01, SNI +LIDO+SMS vs. SNI +NX+SMS ****P* <0.001).

**Appendix S2. SMS induced analgesia in female rats with SNI.**

(A) Paw withdrawal threshold (g) (F (1, 62) =123). (B) Latency to withdrawal (s) (F (1, 62) =123). Two-way ANOVA with Tukey’s multiple comparisons test, n = 8 rats/group,*****P* < 0.0001, SNI +SMS vs. SNI+SHAM SMS.

**Appendix S3. Behavioral changes in rats between SMS and SHAM SMS groups.**

(A) The total number of paw-withdrawal in SMS and SHAM SMS rats.(B) The total number of paw licking in SMS and SHAM SMS rats.(C) The total number of grooming in SMS and SHAM SMS rats.(D) Total distance traveled in the open field test (OFT) for SMS and SHAM SMS rats.(E) Time spent in the central area of the open field in SMS and SHAM SMS rats.(F) Distance traveled in the central area of the open field in SMS and SHAM SMS rats. Paired t test was used for data analysis, n = 5 per group. SMS vs SHAM SMS,*** *p* < 0.001. Representative movement trajectories of (E) SMS group rats and (F) SHAM SMS group rats.

**Appendix S4. Specific activating effects of SMS on NAc Shell GABAergic neurons revealed at the single-cell level**

(A) Schematics illustrating the placement of recording wire tips for NAc Shell-implanted animals. (B) Representative waveform used for analysis of action potential width of putative GABA-ergic pyramidal cells. Width measurements were taken as the time between spike valley and the following peak. (C) The estimated proportions of GABAergic interneuron activity in the NAc Shell of LID or control rats were analyzed. Among the 41 neurons in the LID model group, 22 (53.65%) exhibited excitation, 9 (21.95%) exhibited inhibition, and 10 (24.39%) exhibited no response. In the control group with 42 neurons, 11 (26.19%) exhibited excitation, 14 (33.33%) exhibited inhibition, and 17 (40.48%) exhibited no response. With a sample size of 83 (>40) and a minimum expected frequency of 11.36 (>5), a chi-square test was performed, resulting in a chi-square value of 6.557 with 2 degrees of freedom and *P* <0.05. (D) Spike-issued sequence diagrams of representative channels of each groups. (E) Single neuron examples of the prevalent response to SMS before (-100 –0 sec) and after SMS (0 –100 sec) in the NAc Shellin LID rats or control rats.

**Appendix S5. GABA receptor antagonist in NAc Shell reverses the effects of SMS in LID rats.**

(A) Schematic showing the protocol of cannula implantation and CPP experiment. (B) The effect of SMS on mechanical allodynia threshold in LID rats after microinjection of GABA antagonist Bicuculline (n = 5 in each group; two-way RM ANOVA was used for data analysis; F (24, 128) = 5.295, 0h, LID+SMS+DMSO vs. LID+SMS+Bic ***P* <0.01; 2h, LID+SMS+DMSO vs. LID+SMS+Bic **P* <0.05). (C) The effect of SMS on Paw withdrawal latency threshold in LID rats after microinjection of GABA antagonist Bicuculline (n = 6 in Control+Bic group, LID+SMS+DMSO and LID+SMS+BIC group, n = 5 in LID+SHAM SMS+Bic group; two-way RM ANOVA was used for data analysis; F (12, 76) = 11.56, 0h, LID+SMS+DMSO vs. LID+SMS+Bic ***P* <0.01; 2h, LID+SMS+DMSO vs. LID+SMS+ Bic ***P* <0.01). (D) CPP scores of animals in each group (n =5 in Control+Bic and LID+SHAM SMS+Bic group, n =6 in LID+SMS+DMSO and LID+SMS+Bic group; one-way ANOVA was used for data analysis; F = 7.773, ***P* <0.01, **P* <0.05). (E) Representative activity trajectories of Real-time movement traces among the groups during CPP test.

**Appendix S6. Inactivating IL–NAc Shell pathway reserves SMS-induced analgesia and CPP behaviors.**

(A)Schematic diagram showing the timeline of experiment and bilateral injection rAAV-EF1a-DIO-hM4D(Gi)-EGFP-WPREs or rAAV-EF1a-DIO-EGFP-WPRE-hGH polyA into the IL and rAAV-CaMKlla-CRE-WPRE-hGH PA into the NAc Shell. Only mice with virus expression confined to IL were included in the study (n=6 each group). (B) Fluorescent photomicrographs of representative injection sites in the IL (left) and NAc Shell (right). (C)-(E) The increased thresholds of mechanical allodynia and thermal hyperalgesia and CPP scores induced by SMS were inhibited by CNO intraperitoneal injection. Paw withdrawal threshold (C); Latency to withdrawal (D); CPP scores (E). Data were presented as mean ± s.e.m. The results were analyzed using repeated measures ANOVA with Tukey’s test for post hoc pairwise comparisons. ***P* < 0.01, ****P* < 0.001, DIO-SMS vs DIO-hM4di (Post-CNO/SMS) (n=6 each group).

[**Video S1**](https://submission.wiley.com/api/rex/v2/submissionupload/tenant/1/submission/febe0972-3a49-4014-8781-7a12c068c148/content/b492af20-aedc-4250-b558-58df3b21d583/download) demonstrates the administration of SMS and the subsequent behavioral responses in SNI rats.

[**Video S2**](https://submission.wiley.com/api/rex/v2/submissionupload/tenant/1/submission/febe0972-3a49-4014-8781-7a12c068c148/content/938506c3-1c19-427f-b6a2-710c8f487d28/download) demonstrates the administration of sham SMS and the subsequent behavioral responses in SNI rats.

[**Video S3**](https://submission.wiley.com/api/rex/v2/submissionupload/tenant/1/submission/febe0972-3a49-4014-8781-7a12c068c148/content/666592ad-16bb-4ebf-b01f-5f55ebdcfde7/download) illustrates the spontaneous activity of SNI rats after receiving SMS.
